# Designing the bioproduction of Martian rocket propellant via a biotechnology-enabled in situ resource utilization strategy

**DOI:** 10.1038/s41467-021-26393-7

**Published:** 2021-10-25

**Authors:** Nicholas S. Kruyer, Matthew J. Realff, Wenting Sun, Caroline L. Genzale, Pamela Peralta-Yahya

**Affiliations:** 1 School of Chemical & Biomolecular Engineering, Georgia Institute of Technology, Atlanta, GA 30332 USA; 2 School of Aerospace Engineering, Georgia Institute of Technology, Atlanta, GA 30332 USA; 3 School of Mechanical Engineering, Georgia Institute of Technology, Atlanta, GA 30332 USA; 4 School of Chemistry and Biochemistry, Georgia Institute of Technology, Atlanta, GA 30332 USA

**Keywords:** Industrial microbiology, Metabolic engineering, Computational models, Applied microbiology, Renewable energy

## Abstract

Mars colonization demands technological advances to enable the return of humans to Earth. Shipping the propellant and oxygen for a return journey is not viable. Considering the gravitational and atmospheric differences between Mars and Earth, we propose bioproduction of a Mars-specific rocket propellant, 2,3-butanediol (2,3-BDO), from CO_2_, sunlight and water on Mars via a biotechnology-enabled in situ resource utilization (bio-ISRU) strategy. Photosynthetic cyanobacteria convert Martian CO_2_ into sugars that are upgraded by engineered *Escherichia coli* into 2,3-BDO. A state-of-the-art bio-ISRU for 2,3-BDO production uses 32% less power and requires a 2.8-fold higher payload mass than proposed chemical ISRU strategies, and generates 44 tons of excess oxygen to support colonization. Attainable, model-guided biological and materials optimizations result in an optimized bio-ISRU that uses 59% less power and has a 13% lower payload mass, while still generating 20 tons excess oxygen. Addressing the identified challenges will advance prospects for interplanetary space travel.

## Abstract (web summary)

Returning from Mars to Earth requires propellant. The authors propose a biotechnology-enabled in situ resource utilization (bioISRU) process to produce a Mars specific rocket propellant, 2,3-butanediol, using cyanobacteria and engineered *E. coli*, with lower payload mass and energy usage compared to chemical ISRU strategies.

## Introduction

**Fig. 1:**
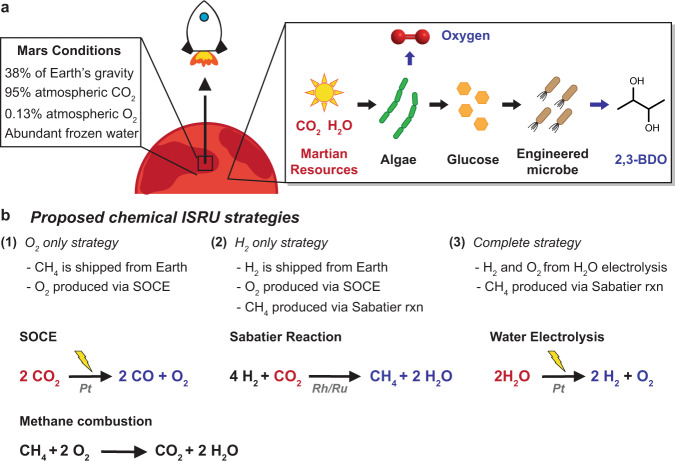
Rocket propellant production on Mars via in situ resource utilization (ISRU). **a** Proposed biotechnology-enabled ISRU (bio-ISRU) takes into account Martian conditions to produce a Mars-specific propellant, 2,3-butanediol (2,3-BDO). Using sunlight, carbon dioxide (CO_2_), and water, cyanobacteria (algae) are grown as a feedstock for an engineered microbe that produces 2,3-BDO. Cyanobacteria cultivation evolves oxygen for spacecraft launches or other aspects of Martian exploration. **b** Proposed chemical ISRU strategies focus on the use of methane (CH_4_) as a propellant. The most examined chemical processes that can be part of a chemical ISRU strategy^2^. (1) O_2_ only strategy. SOCE: solid oxide CO_2_ electrolysis. (2) H_2_ only strategy. (3) Complete strategy. Key chemical reactions: SOCE, Sabatier reaction, and water electrolysis. Martian resources in red. Chemicals produced on Mars in blue. Chemical shipped from Earth in black. Details on proposed biological and chemical ISRU strategies can be found in Table 1.

The success of the NASA Perseverance mission proves that the age of Martian exploration is upon us. For human presence beyond Earth to become a reality^1^, we must not only send humans far into space but also safely bring them back home. A key challenge for this vision is the origin of the fuel needed to bring spacecraft back to Earth. Transporting from Earth to Mars the ~30 tons of methane and liquid oxygen (LOX) needed to power a launch of a human Mars Ascent Vehicle (MAV) from the surface of Mars into low Mars orbit would require an initial payload of 500 tons, and would cost ~$8 billion to launch from Earth. A biotechnology-enabled in situ resource utilization (bio-ISRU) strategy would leverage Martian conditions, including low gravity, abundant CO_2_, water, and access to sunlight, to produce a Mars-specific rocket propellant on Mars (Fig. 1a). In addition to reduced launch mass, ISRU strategies can reduce Mars lander mass and eliminate safety risks associated with the transportation of large stocks of rocket propellant.

Proposed ISRU strategies for rocket propellant production on Mars, including the NASA Human Exploration of Mars Design Reference Architecture (DRA) 5.0, focus on methane (CH_4_) as an alternative propellant due to its favorable heating value and specific impulse (a measurement of rocket performance per unit mass of propellant, *I*_sp_) compared to rocket propellant 1 (RP-1)^2^. To produce CH_4_ and O_2_ on Mars, the DRA 5.0 relies on up to three chemical processes: (1) solid oxide carbon dioxide (CO_2_) electrolysis (SOCE) to convert Martian CO_2_ to O_2_, (2) the Sabatier reaction to convert hydrogen (H_2_) and CO_2_ into CH_4_, and (3) water electrolysis to produce H_2_ and O_2_ (Fig. 1b). Of note, production of O_2_ on Mars is critical, as Martian O_2_ levels (0.13%, 9 × 10^−3^ hPa) are 20,000 times lower than on Earth (210 hPa), making them insufficient for rocket propellant burning^3^. The DRA 5.0 proposes three chemical ISRU strategies based on their technology readiness level (Fig. 1b, Table 1). The most likely initial strategy is the DRA 5.0 O_2_ only strategy, wherein CH_4_ is shipped from Earth and O_2_ is produced on Mars via SOCE. The two other strategies rely on shipping H_2_ from Earth, or producing H_2_ on Mars via water electrolysis, and using the Sabatier reaction to convert H_2_ to CH_4_. Oxygen for combustion would be produced via SOCE or H_2_O electrolysis. Practically, the large H_2_ shipment volume, tripling the size of the delivery spacecraft, and the need for supplemental O_2_ production through SOCE, as the Sabatier reaction does not produce sufficient O_2_ to burn all the CH_4_ produced, limit these last two chemical ISRU strategies. The DRA 5.0 O_2_ only strategy reduces the payload mass from Earth from 30 tons (for CH_4_ and O_2_) to 7.5 tons, ~6.5 tons of CH_4,_ and ~1 ton of SOCE equipment^2^.

**Table 1:** Comparison of proposed in situ resource utilization (ISRU) strategies for rocket propellant production on Mars.

	Strategy	Propellant	Propellant production (ton)	O_2_ production (ton)	O_2_ for launch (ton)	Excess O_2_ (ton)	Payload (ton)	Power usage (kW)
Chemical ISRU^a^	O_2_ only^b^	CH_4_	N/A	22.98^e^	22.98^e^	0	7.51	26.08
	H_2_ only^c^		6.57				3.25	33.3
	Complete^d^		6.57				2.66	63.77
Bio-ISRU^f^	State-of-the-art	2,3-BDO	10	63.41^g^	19.6	43.81	20.94	17.64
	Optimized			39.95^h^		20.35	6.53	10.80

^a^Chemical ISRU numbers from DRA 5.0 (ref. ^2^).

^b^CH_4_ shipped from Earth. O_2_ produced via solid oxide carbon dioxide electrolysis (SOCE).

^c^H_2_ shipped from Earth. CH_4_ produced via Sabatier reaction. O_2_ produced via SOCE.

^d^H_2_ and O_2_ produced via water electrolysis. CH_4_ produced via Sabatier reaction.

^e^O_2_ needed according to DRA 5.0, which uses a 3.5:1 O_2_/methane ratio rather than the 4:1 ratio needed for full combustion (26.27 tons of O_2_). The actual O_2_ value is between 3.5 and 4.

^f^Biotechnology-enabled ISRU process-Biofilm: 2,3-BDO produced on Mars from CO_2_, H_2_O, and sunlight.

^g^Number already takes into account the 12 tons needed for 2,3-BDO production (fermenter).

^h^Number already takes into account the 7.6 tons needed for 2,3-BDO production (fermenter).

**Fig. 2:**
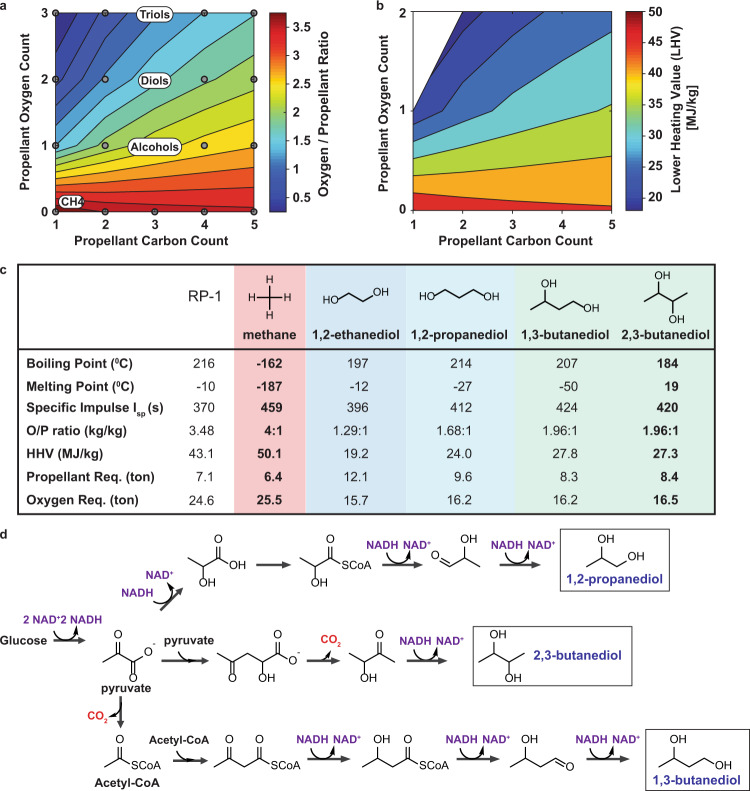
Martian rocket propellant design. **a** Relationship between increasing the number of oxygen and carbon atoms in the propellants to the mass of oxygen needed for complete combustion, i.e. oxygen/propellant ratio (O/P ratio). Increasing the number of oxygen atoms in the propellant reduces the mass of external oxygen needed for full combustion. **b** Relationship between increasing the number of oxygen and carbon atoms in the propellant to its lower heating value (LHV). Increasing the number of oxygen atoms in the propellant reduces its LHV. **c** Propellant relevant properties for Rocket Propellant-1 (RP-1), used for spacecraft launch from Earth, methane, and proposed Martian rocket propellants: 1,2-ethanediol, 1,3-propanediol, 1,3-butanediol, and 2,3-butanediol. Theoretical specific impulses (*I*_sp_) were calculated using Eq. (3). Mass of propellant required was calculated using the ideal rocket equation (Eq. (4)). **d** Short-chain diol metabolic pathways from glucose. Both 1,2-propanediol and 1,3-butanediol require excess NADH. Carbon dioxide evolved: red. NADH consumed: purple. Name of short-chain diols: blue. Source data underlying Fig. 2c are provided as a Source Data file.

ISRU strategies for rocket propellant production on Mars should consider the differences in gravity and atmospheric composition between Mars and Earth when designing alternative propellants. Mars has 38% of Earth’s gravity (3.73 and 9.81 m/s^2^, respectively)^3^, thus Martian rocket propellants have a lower energy density requirement. The low O_2_ levels in the Martian atmosphere also impact rocket propellant design, as the amount of O_2_ needed for fuel-burning depends on the propellant’s chemical composition. Non-oxygen containing compounds require more LOX than oxygen-containing compounds (Fig. 2a). For example, while CH_4_ requires a 4:1 oxygen to propellant mass ratio, methanol (CH_3_OH) requires a 1.5:1 ratio for complete combustion. Although the presence of oxygen atoms lowers the heating value of fuel (Fig. 2b), Mars’ lower gravity allows some of these chemicals to be feasible as rocket propellants. Further, theoretical *I*_sp_ values for oxygenated chemicals, in particular short-chain diols, are similar to *I*_sp_ values for methane (CH_4_ *I*_sp_ = 459 s; 2,3-BDO *I*_sp_ = 420 s), rendering these chemicals feasible as Martian rocket propellants without sacrificing engine performance for the reduced O_2_ requirement (Fig. 2c, Eqs. (1)–(3)).

Biological systems can convert CO_2_ into chemicals, and Mars has 20 times more CO_2_ than Earth (6.67 and 0.38 hPa, respectively), accounting for ~95% of total atmospheric pressure, offering an excellent carbon source for hydrocarbon rocket propellant production. Indeed, biotechnology-enabled ISRU strategies that leverage autotrophs have been proposed for the production of fuels, materials, and astronaut nutrition^4^. For example, methanogens convert CO_2_ to CH_4_ at 96% purity with a productivity of 530 mmol/L/h^5^. Using this system, a 5.66 L reactor provides the same hourly CH_4_ productivity (48 g h^−1^) as the ~1.5 L Sabatier reactor currently in use on the International Space Station^6^. Photosynthetic cyanobacteria convert CO_2_ and sunlight to biomass and chemicals^7^ evolving O_2_ as a byproduct, which, unlike SOCE generated O_2_, is not mixed with carbon monoxide. Nevertheless, the assortment and yields of chemicals produced in cyanobacteria are orders of magnitude smaller than those achieved in biosynthetic workhorses, such as *Escherichia coli* and *Saccharomyces cerevisiae*, which have been more extensively engineered for chemical production^7,8^.

Rather than having autotrophs capture CO_2_ and upgrade it to a rocket propellant, the autotroph can be grown as a feedstock for an engineered heterotrophic microbe to produce the propellant (Fig. 1a). *S. cerevisiae* can produce ethanol from cyanobacteria biomass without any additional nutrients^9,10^. *E. coli* can produce 5.9 g/L of alcohols with four to five carbons with 57% yield from cyanobacterial sugars and 32% yield from cyanobacterial proteins^11^. Additionally, the shorter doubling time of heterotrophs (*S. cerevisiae*: 3 h, *E. coli*: 0.5 h) when compared to autotrophs (cyanobacteria: 7 h) enables a process with smaller cyanobacteria farms together with smaller and shorter fermenter production runs to synthesize the rocket propellant. After propellant extraction, the heterotroph’s biomass could be fed to the autotroph, maximizing nutrient reuse.

Here, we propose a biologically enabled ISRU strategy (bio-ISRU) to produce a Mars-specific rocket propellant, 2,3-butanediol (2,3-BDO), from sunlight, CO_2_, and Martian water (Fig. 1a). Specifically, cyanobacteria capture Martian CO_2_ and convert it to simple sugars and key nutrients, which an engineered *E. coli* converts to 2,3-BDO in a continuous process. Key to this analysis are the constraints imposed by the Martian setting, such as light intensity and water limitations. Our process model includes (1) the continuous cultivation of cyanobacteria; (2) the enzymatic digestion of cyanobacteria biomass to recover sugars, nitrogen and trace nutrients; (3) the cultivation of *E. coli* for 2,3-BDO production from cyanobacterial glucose; and (4) the separation and purification of 2,3-BDO from the fermentation broth to 95% purity. When compared to the proposed DRA 5.0 O_2_ only strategy, the bio-ISRU strategy for 2,3-BDO production using existing state-of-the-art technology requires a 2.8-fold higher payload mass from Earth, yet consumes 32% less power while generating 44 tons of excess clean O_2_. The excess O_2_ can be used for subsequent spacecraft launches or other aspects of Martian exploration, such as human colony formation, offering a distinct advantage over the chemical system. To render the bio-ISRU competitive with the DRA 5.0 O_2_ only strategy in terms of payload mass from Earth, our process model identifies key biological and materials optimizations. Biological improvements in cyanobacterial biomass productivity, enzymatic digestion of cyanobacterial biomass, and *E. coli* 2,3-BDO yield, together with the use of lighter-weight process materials, result in an optimized bio-ISRU with 13% lower payload mass and 59% lower power consumption than the DRA 5.0 O_2_ only strategy, while still generating 20 tons of excess oxygen. Further, subsequent bio-ISRU missions will require 3.73 tons resupply payload mass, almost 50% less than the 6.5 ton payload mass for subsequent DRA 5.0 O_2_ only strategy missions. Taken together, the biological production of rocket propellant on Mars is within reach using state-of-the-art technology. The proposed bio-ISRU strategy also has the key advantage of producing oxygen. Addressing the identified biological and materials challenges should take us closer to enabling interplanetary space travel and Mars colonization.

## Results

### Challenges in producing rocket propellant on Mars

While chemical bioproduction unit operations are similar on Mars and on Earth, special considerations must be made for implementation on Mars. First, the average Martian surface temperature is −55 °C compared to 15 °C on Earth^3^. While temperatures as low as −60 °C do not affect microbial viability, microbes do not grow at those temperatures. Our analysis assumes a 25 °C process temperature. Although not optimal for microbial growth (cyanobacteria: 35 °C; *E. coli*: 37 °C), it allows reasonable growth rates. Temperature control is a crucial assumption for our process design, though it remains an unsolved challenge. It could be achieved via reactor jacketing, use of a transparent dome-like structure to trap radiant solar heat akin to a greenhouse^12^, temperature control systems, or some combination thereof^13^. Second, the thinner Martian atmosphere results in higher ionizing and gamma radiation levels reaching the surface as compared to Earth. Fortunately, many cyanobacteria, including *Arthrospira platensis*, and *E. coli* are resistant to Martian radiation levels, maintaining viability for centuries^14,15^. Of more concern is the level of UV radiation that reaches the Martian surface, which poses a risk for genetic mutations. Thus, bags for cyanobacteria growth and/or the greenhouse dome should be made of a UV reflecting material while allowing transmission of photosynthetically active radiation (PAR). For example, flexible polymers, including polyvinyl alcohol or poly(dimethyl)siloxane doped with UV absorbent nanoparticles (ZrO_2_, SiO_2_^16^) or chemicals (sepia eumelanin^17^). Importantly, the PAR intensity that reaches the Martian surface is 57% lower than on Earth due to the increased distance from the sun^3^. This decrease in photon flux will result in a decreased cyanobacteria photosynthetic growth rate, thus biomass productivity is modeled using light as the growth-limiting factor. Further, frequent Martian dust storms can disrupt photon flux, and reduce photosynthetic rates. Prior to the implementation of a photosynthetically driven process, detailed analysis of the mission landing site can help predict the frequency and severity of the dust storms to inform changes to the model^18^. Third, biological processes require water. Although recent reports suggest that Martian water exists in sufficient amounts for the proposed bio-ISRU, harvested water may need to be pretreated to reduce salt content to enable microbial growth^19^. Fourth, microbial growth requires nitrogen, phosphorus, and trace metals in addition to carbon. While some trace metals (e.g. Mg, Ca, K and Na) could be obtained from Mars in a bioavailable form via electrochemistry, to mitigate uncertainty and the presence of perchlorates in Martian regolith^20^, the majority of these nutrients will need to be shipped from Earth^21^. Of note, trace metals fed to the cyanobacteria will carry through to the hydrolysate fed to *E. coli*^22^. To complete the cycle, *E. coli* biomass can be fed back to the cyanobacteria after rocket propellant separation to effectively recycle the trace metals and nitrogen^23^. Fifth, Mars has a nitrogen (N_2_) partial pressure of 0.189 hPa^3^, 10-fold lower than that required for cyanobacterial N_2_ fixation^24^. In our analysis, we have not considered N_2_ fixation, and use a cyanobacteria, *A. platensis*, that does not fix N_2_. Instead, we propose shipping diammonium phosphate ((NH_4_)_2_PO_4_) and ammonia (NH_3_) from Earth. While implementation of N_2_ fixing cyanobacteria, such as *Anabaena cylindrica*, would reduce nutrient payload mass, it would likely require atmospheric N_2_ concentration or cyanobacteria engineering to enable cyanobacterial N_2_ fixation^3^. Finally, the entire infrastructure, including nuclear power sources, must be shipped from Earth. This has direct implications for reactor design and whole process design as payload mass directly impacts mission cost. To account for these challenges, the proposed bio-ISRU analyzes water use, power requirements, and total infrastructure payload mass as key process metrics.

### Martian rocket propellant design

Based on energy density, phase behavior, and biological reachability, we propose C_3_–C_4_ hydrocarbons with two oxygen atoms (diols) as potential Martian rocket propellants. The oxygen content within the propellant structure reduces the amount of LOX needed for combustion, i.e. the stoichiometric O_2_/propellant mixture ratio (Fig. 2a). A lower ratio is advantageous on Mars due to its low atmospheric O_2_ content, making oxygen-containing compounds (e.g. alcohols, diols) preferable for Martian application. Compared to alcohols and C_1_–C_2_ diols, C_3_–C_4_ diols have the required heating value (LHV, 20–25 MJ/kg) and specific impulse (*I*_sp_, ~400 s) to propel a human Mars Ascent Vehicle (MAV) with lower oxidizer need (Fig. 2b). Triols require less oxidizer, but their LHV is too low to propel a human MAV. Diols require less than half the LOX mass per mass of fuel when compared to methane. Additionally, C_3_–C_4_ diols have appropriate boiling and melting points to remain liquid or solid over Martian temperature (−153 to 20 °C), and thus have lower volumetric and energetic storage requirements (Fig. 2c). Therefore, the energy needed to compress, or refrigerate, gaseous methane to a liquid for storage and propulsion is sidestepped. Finally, with respect to bioreachability, several natural and engineered microbes produce C_3_–C_4_ diols in high titers and yields^25^, whereas C5 diols production is low^26^, and >C5 diols production has been elusive. In particular, we consider 1,2-propanediol (1,2-PDO) (C_3_)^27^, 1,3-butanediol (1,3-BDO) (C_4_)^28^, and 2,3-BDO (C_4_)^29^ as Martian rocket propellants, all of which can be biologically synthesized today (Fig. 2d).

### 2,3-BDO as a Mars-specific rocket propellant

To assess the feasibility of each of the three diols, we calculate the *E. coli* maximum theoretical yield from glucose using the Constraint-Based Reconstruction and Analysis (COBRA) toolbox^30^ and the iML1515 *E. coli* genome-scale model^31^. Theoretical yields for 1,2-PDO, 1,3-BDO, and 2,3-BDO are 0.615, 0.543, and 0.538 g diol/g glucose, respectively. Although 1,2-PDO and 1,3-BDO have the highest theoretical yields, the yields are limited by NADH requirements. While 1 mol of 2,3-BDO generates one mol of NADH, one mole of 1,2-PDO or one mol of 1,3-BDO results in the net consumption of one mol of NADH (Fig. 2d). NADH is a redox balance cofactor required by many cellular processes, and higher NADH demand often results in reduced cell growth and carbon flux through the desired pathway. Furthermore, NADH generation relies on O_2_ during glycolysis and thus can be limited by poor O_2_ transport at an industrial scale.

Among the three diols, 2,3-BDO is at the highest technology readiness level, produced in *E. coli* at high titer (73.8 g/L), yield (0.432 g/g glucose), and productivity (1.17 g/L/h)^29^. That is, 2,3-BDO is produced at 80% of the theoretical maximum. The experimental yield of 2,3-BDO is 2.4 times higher than that of 1,2-PDO (0.178 g/g glucose)^27^ and 3.9 times higher than that of 1,3-BDO (0.11 g/g glucose^28^). For these reasons we focus our analysis on 2,3-BDO, which will lead to a smaller cyanobacteria farm footprint, microbial fermenter size, and fewer fermentation byproducts, streamlining 2,3-BDO purification. Additionally, separation processes for 2,3-BDO from fermentation broth are well established^32^ whereas separation processes for 1,2-PDO and 1,3-BDO are still being explored.

To set the 2,3-BDO mass production target for a 500-day human surface visit to Mars mission, we applied the ideal rocket equation using the theoretical *I*_sp_ value and the oxygen fuel ratios for 2,3-BDO (Eq. (4)). As there are no experimental *I*_sp_ values for diols, we calculated the theoretical *I*_sp_ of 2,3-BDO to be 420 s. A total of 8.4 tons of 2,3-BDO and 16.5 tons of LOX is required to power a MAV. The total required propellant plus LOX is 18% lower when using 2,3-BDO instead of methane (Table 1). Of note, the theoretical *I*_sp_ values for methane and 2,3-BDO are in the same range (~400 s). Importantly, as the literature value for CH_4_ *I*_sp_ is ~369 s^2^, it is possible that 2,3-BDO has a lower experimental value. Therefore, we have increased the 2,3-BDO mass production target estimate to 10 tons, which assumes a very conservative 2,3-BDO *I*_sp_ of 383 s. The presented process metrics are based on producing 10 tons of 2,3-BDO, requiring 19.6 tons of LOX.

### Bio-ISRU for 2,3-BDO process design overview

**Fig. 3:**
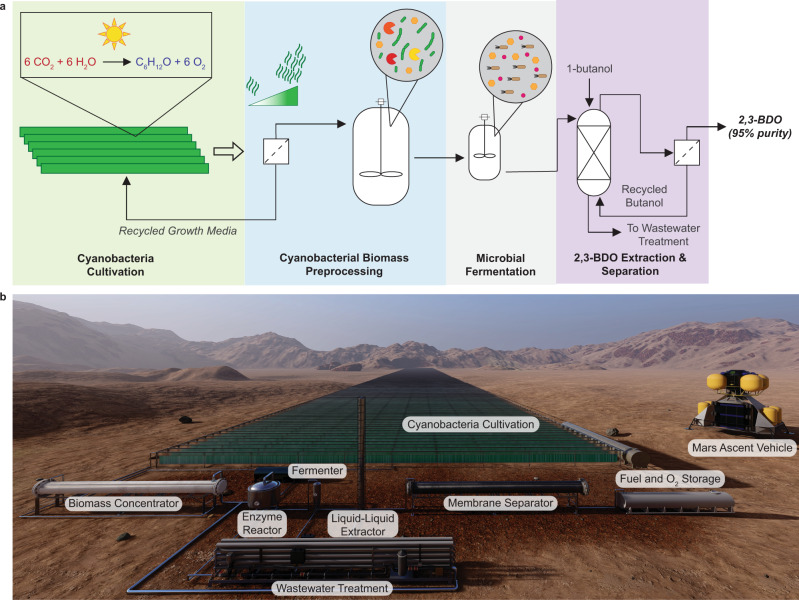
Continuous bio-ISRU production of 2,3-BDO on Mars. **a** The bio-ISRU production of 2,3-BDO is composed of four modules: (1) Cyanobacterial cultivation in photobioreactors or biofilm growth (shaded green), (2) Cyanobacteria biomass preprocessing composed of biomass concentration via membrane filtration and enzymatic digestion in a stirred tank (shaded blue), (3) Microbial fermentation of the cyanobacterial glucose to produce 2,3-BDO (shaded gray), (4) 2,3-BDO extraction and separation via sequential liquid–liquid extraction and membrane separation to achieve 95% purity (shaded purple). Chemical formula: Red Martian resources. Blue chemicals made on Mars. **b** Rendering of what the bio-ISRU for 2,3-BDO process might look like on Mars, with a Mars Ascent Vehicle included for scale. The cyanobacterial cultivation module makes up the majority of the material and land footprints.

The bio-ISRU for 2,3-BDO is composed of four modules: (1) cyanobacteria cultivation using Martian CO_2_ and sunlight, (2) cyanobacteria biomass preprocessing, consisting of biomass concentration and enzymatic digestion to release sugars and trace nutrients for use by the heterotrophic microbe, (3) microbial fermentation to upgrade the sugars into 2,3-BDO, and (4) 2,3-BDO extraction and separation to ~95% purity from the microbial broth (Fig. 3).

### Cyanobacteria growth

To fix Martian CO_2_, we will use *A. platensis* (i.e. spirulina), which under nitrogen limitation maximizes glycogen production, up to 60% of dry cell weight (DCW)^9^. *A. platensis* grows well at 0.38 hPa of CO_2_, thus the 6.67 hPa CO_2_ present on Mars will enable robust *A. platensis* growth. Indeed, higher CO_2_ levels improve cyanobacteria growth^33^. Beyond CO_2_ concentration, a process concern is the negative effect of the <10 hPa total atmospheric pressure on Mars versus 1013 hPa on Earth. Elevated CO_2_ levels should help to mitigate this effect, with 50 hPa of CO_2_ in a 100% CO_2_ atmosphere showing uninhibited growth^34^. However, the Martian atmosphere still has a 5-fold lower pressure than previously studied. Thus, CO_2_ pressurization may be required to achieve optimal growth, and further study at these low pressures is needed to determine the full effects and validate our assumption that growth is indeed light limited and not CO_2_ limited.

Cyanobacteria culturing requires the addition of nitrogen and phosphorus, as well as trace elements (Ca, Cu, Fe, K, Mg, Mn, Na, and Zn)^35^. To reduce mission risk, all the required nitrogen and phosphorus to produce enough cyanobacterial biomass to reach the 15 tons of 2,3-BDO mass target will be shipped from Earth. Required nitrogen and phosphorus mass were calculated based on the elemental composition of cyanobacteria (C_4.5_H_8.2_N_0.129_O_2.219_S_0.006_P_0.007_ ref. ^36^). Nutrients will be provided in 20% excess to enable adequate uptake concentrations in the growth media^37^. Under these requirements 0.75 tons of (NH_4_)_2_PO_4_, and 1.20 tons of anhydrous NH_3_ are included in payload mass calculations. Alternative sources of nitrogen and phosphorus include fixing N_2_ in situ via sunlight-driven chemical catalysis^38^ or harvesting phosphorus from Martian regolith^39^. However, regolith harvesting requires additional time and infrastructure. Further, the harvested regolith contains harmful perchlorates^20^, and the nutrients may not be readily dissolvable for microorganism consumption. Trace elements are provided based on their previously reported minimum requirements to support unhindered cyanobacterial growth and are recycled through the process^35^.

### Cyanobacteria cultivation

**Fig. 4:**
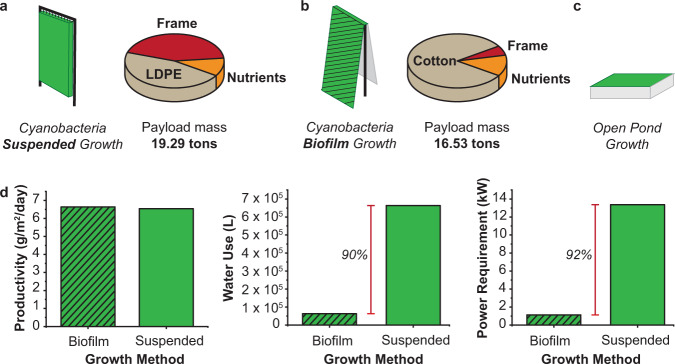
Cyanobacteria cultivation unit metrics required for the bio-ISRU production of 2,3-BDO. Schematic of cyanobacteria growth systems (green) and payload mass broken down into nutrients (orange), frame (red) and material (brown): **a** Suspended growth: cyanobacteria grown in photobioreactors (0.3 mm-thick low-density polyethylene, LDPE) filled with water; **b** Biofilm growth: cyanobacteria grown in a thin film on a porous substrate (0.3 mm thick cotton fabric); **c** Open pond: cyanobacteria grown open to the atmosphere. Not evaluated for use on Mars. **d** Cyanobacteria biomass productivity, water use and power requirement for biofilm and suspended growth. Results are based on 10 tons of 2,3-BDO produced over 500 sols. Source data underlying **a**, **b**, and **d** are provided as a Source Data file.

While Martian water is sufficient to sustain the bio-ISRU, harvesting and keeping it in liquid form will require 1–2 kW per ton of water^2^. With this in mind, we explored two cyanobacteria cultivation methods: water-intensive suspended growth and less water-intensive biofilm growth. We did not explore an open pond strategy due to contamination of the Martian environment and water evaporation concerns. (Fig. 4a–c).

For the suspended cyanobacteria growth model, we use low density polyethylene (LDPE) photobioreactors (PBRs)^40^, which lower contamination threats to the Martian environment, have low evaporation rates, allow highly controllable growth rates through mixing, enable CO_2_ delivery via gas bubbling, and can be shipped in rolls to be inflated upon arrival. On Earth, suspended growth is used for the pilot- and demonstration-scale production of cyanobacterial biomass for food and biodiesel^36^. Suspended growth biomass productivity was modeled using Monod growth kinetics based on lab- and pilot-scale growth of *A. platensis* on Earth^41^ with key variations to take into account Martian conditions. The model assumes that on Mars, as it is the case on Earth^41^, cyanobacteria growth is limited by light penetration rather than CO_2_, nitrogen, or phosphorus sources. Key model variations to account for Martian conditions include: (1) Photon flux at the reactor surface (*E*_0_), which takes into account the 57% lower photon flux on Mars than on Earth^3^, (2) Reactor diameter (*D*), which was optimized to achieve maximal light penetration on Mars, by spacing the 4.5 cm wide PBR units 1 m apart^36^, and (3) photon flux (*E*_K_) and cellular respiration rate (*R*_o_) at 25 °C. Hanging bag PBRs rather than a horizontal system was modeled to increase the ratio of light absorption area, i.e. PBR surface area to land area (*F* ratio: 1.84; horizontal system *F* ratio: 1). The *F* ratio could be increased to 15 by reducing the PBR spacing or increasing the PBR height, at the expense of reduced light intensity at the PBR surface due to shading from surrounding bags^42^. As on Earth, biomass concentration is modeled at 1 g/L to allow for maximum light penetration into the culture. All other parameters were taken from literature^41^, resulting in a modeled biomass productivity of 6.54 g/m^2^/day. This biomass productivity is given in terms of land area footprint of the cyanobacteria farm to enable the use of published power and mass correlations^36^. The cyanobacterial cultivation will be run as a continuous reactor where the calculated biomass productivity was used to determine the rate of constant biomass harvest to maintain the culture at the desired concentration of 1 g/L. The calculated productivity requires processing of 0.105 tons (105 kg) of biomass per day to reach our production goal and timeline. On Earth, cyanobacteria have growth productivity between 20 and 30 g/m^2^/day at temperatures from 20 to 30 °C for both suspended and biofilm growth^36,43^.

Cyanobacteria biofilm growth holds promise in improved gas delivery, light penetration, reduced water usage, and reduced energy demand due to the elimination of culture mixing. Biofilm growth has been performed at pilot but not industrial scale^43^. Due to the density of the biofilm and reduced water flow^42^, it has a lower contamination risk than suspended growth. Indeed, large-scale, outdoor, biofilm cultivation experiments have avoided contamination without implementation of special measures^43–45^. If this scenario were not extended to our system, contamination risk of the open biofilm system could be mitigated by using a cyanobacteria extremophile, such as the salt-tolerant *Tolypothrix* sp.^46^. Cyanobacteria biofilm growth was modeled using the same growth model used for suspended growth. Biofilm will be grown on a hydrophilic, porous, growth substrate^47^, resembling a cotton towel^43^, 0.3 mm thick to match the thickness of the LDPE PBR bags^40^. Only light penetration (*k*), reactor diameter (*D*), i.e. film thickness, and areal chlorophyll concentration (*C*_o_), which depend on the increased biomass concentration in biofilm growth (7.5 g/m^2^ versus 1 g/L) were changed. Other growth parameters, including photosynthetic efficiency, respiration rate, and optimal photon flux, were assumed to be species-dependent, and thus independent of growth method and were used unchanged. For direct comparison, the 1 m spacing and *F* ratio of 1.84 were also applied to biofilm growth, resulting in a modeled productivity of 6.64 g/m^2^/day, similar to the value modeled for suspended growth.

Suspended and biofilm growth result in similar biomass productivities (~6.6 g/m^2^/day); however, biofilm growth uses 90% less water (6.33 × 10^4^ L) when compared to suspended growth (6.64 × 10^5^ L)^45^ to produce 52.4 tons of cyanobacteria biomass over 500 sols (Fig. 4d). Based on water harvesting via soil evaporation^2^, biofilm growth results in energy savings of up to 1200 kW. Further, the biofilm system payload mass is 14% less than that of the suspended system. The reduction is due to the mass of the suspended growth frame needed to hold the PBR bags, even considering the reduced system weight under Martian gravity (Fig. 4a, b). Of note, the cotton material used as the biofilm growth substrate accounts for 82% of the biofilm growth method mass (Fig. 4a).

### Cyanobacteria biomass concentration

Cross-flow filtration was modeled to concentrate cyanobacteria due to its lower payload mass when compared to centrifugation equipment, and more rapid and efficient separation than settling tanks^48^. A hydrophilic and neutrally charged membrane concentrates the biomass from 1 g/L in the PBR to 20 g/L for input into the enzymatic digester. The membrane is modeled to have a 40 kDa cut-off^49,50^ with long-term membrane flux approaching 40 L/m^2^/h and stability of 6 weeks^51^. Biomass collection from biofilm growth was set at a continuous 20 g/L, eliminating the need for a concentration unit operation for biofilm cultivation mode^9^. Biofilm biomass will be harvested using low-energy mechanical scraping^52^. While continuous harvesting of biofilm biomass has not been implemented at pilot or industrial scale, approaches for scale-up include rotation of planar^53^ or circular^54^ growth substrate, or use of a high-velocity water flow. A further experimental investigation is required to fully model power and mass requirements for biofilm biomass harvesting, which will be crucial for Martian implementation.

### Cyanobacteria biomass enzymatic digestion

**Fig. 5:**
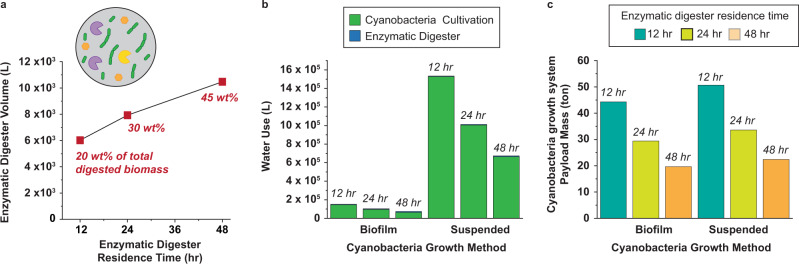
Cyanobacteria biomass preprocessing metrics required for the bio-ISRU production of 2,3-BDO. **a** Weight percent of total cyanobacteria biomass digested into glucose depends on the enzymatic digester residence time. The enzymatic digester size increases with increasing residence time. Inset is a cartoon of the enzyme digester composition: cyanobacteria (green), digesting enzymes (yellow, purple), released sugars (orange hexagons). Increasing the enzymatic digester residence time **b** decreases the volume of water needed and **c** decreases the payload mass of the cyanobacteria growth system. Results are based on 10 tons of 2,3-BDO produced over 500 sols. Source data underlying **b** and **c** are provided as a Source Data file.

Lysozyme, α-amylase, and glucoamylase digestion releases between 0.3 and 0.45 g of glucose/g of cyanobacterial biomass over 24 and 48 h, respectively^9^. A shorter enzymatic digester residence time requires a smaller digester volume and reduced water usage; however, it also requires more cyanobacteria biomass to achieve the desired sugar output, thus a larger cyanobacteria farm. The goal is to convert the 52.4 tons of cyanobacteria biomass to the 23.2 tons of glucose required to produce 10 tons of 2,3-BDO. Increasing biomass digestion from 24 to 48 h reduces cyanobacteria growth system biomass requirement by ~34%, resulting in a ~34% reduction in algae water requirement, and a 33% reduction in payload mass of the cyanobacterial growth system for both biofilm and suspended growth systems (Fig. 5). Taken together, decreasing the size of the cyanobacterial farm is beneficial, even at the expense of increased size of the biomass digester.

### Microbial production of rocket propellant on Mars

*E. coli* was modeled to convert cyanobacterial glucose to 2,3-BDO, as hydrolyzed cyanobacteria have been used as the sole feedstock to drive *E. coli* growth^55^ and chemical production^11^. As Mars is devoid of life, using a non-pathogenic microbe reduces environmental contamination risks, while process implementation could take advantage of the high levels of germicidal UV radiation that reach the surface of Mars to sterilize process components prior to inoculation. *E. coli* has been engineered to produce 2,3-BDO, and for process design we used steady-state productivity of 1.17 g 2,3-BDO/L/h and a yield of 0.432 g 2,3-BDO/g glucose, matching the *E. coli* state-of-the-art^29^. Of note, O_2_ is important for NADH regeneration during 2,3-BDO production^29^. Cyanobacterial cultivation produces excess O_2_ to meet the ~12 tons requirement of the fermentation unit over the course of 500 sols (500 Mars days) in addition to the O_2_ required for rocket propellant (19.6 tons, Table 1).

### Rocket propellant extraction and separation

We modeled liquid–liquid extraction (LLE) followed by membrane-based separation to purify 2,3-BDO from the fermentation broth. In the LLE unit, hydrophilic 2,3-BDO is extracted from fermentation broth using butanol, a previously validated solvent^56^, which does not theoretically reduce fuel performance if not fully removed from 2,3-BDO judging by its similar heating value (36 MJ/kg) and *I*_sp_ (439 s). LLE was modeled using the Phasepy python package^57^ with binary interaction parameters regressed in Aspen Plus (AspenTech). To concentrate and dewater 2,3-BDO, a polydimethylsiloxane/polyvinyledenefluoride membrane was modeled using flux differential equations^56^. Our model indicated that 0.5 m^2^ of membrane area is required to reach 95% 2,3-BDO purity. Thus, the membrane separations unit accounts for <1% of total payload mass. As butanol must be shipped from Earth, the process includes a solvent recycle stream to minimize the overall solvent input into the system. To reduce water usage, the water-rich stream coming out of the LLE unit will be purified in the water recycling unit operation and fed back to the *E. coli* fermenter (Fig. 3). An important consideration will be the amount of butanol lost in this stream, as lost butanol will increase payload mass and introduce toxicity risks to *E. coli*. Our analysis shows that the butanol concentration in water outlet stream will be ~0.01%. Nevertheless, careful consideration must be taken to prevent butanol accumulation to not run into *E. coli* toxicity. To mitigate this risk, *E. coli* could be engineered for improved butanol tolerance^58^.

### Water recycling

Martian water will be sufficient to support a Martian colony as well as the bio-ISRU of 2,3-BDO^19^. However, maximization of process efficiency and integration requires wastewater management and recycling. Implementation of reverse osmosis for wastewater purification will prevent the buildup of salts and proteins over the course of the process. The concentrated waste stream can be reintroduced to the process in order to recycle trace nutrients, while the remainder of the rejected waste can be utilized in other ISRU applications, such as for fertilizer^59^ or for astronaut nutrition^60^, as the bio-ISRU does not produce toxic compounds. For this application, a reverse osmosis membrane with long-term stability and a lightweight material was modeled. Specifically, a thin-film composite membrane, 200 µM thick with water flux of 25 L/m^2^/h^61^. Based on the water-rich stream flow rate of 3.64 L/min coming out of the LLE unit, a membrane area of 8.73 m^2^ is required, bringing the mass of the required water treatment unit to 0.008 tons, 0.03% of total payload mass. Importantly, this takes into account the mass of the membrane and housing, but not any required pumps and control systems.

### Bio-ISRU full process analysis

**Fig. 6:**
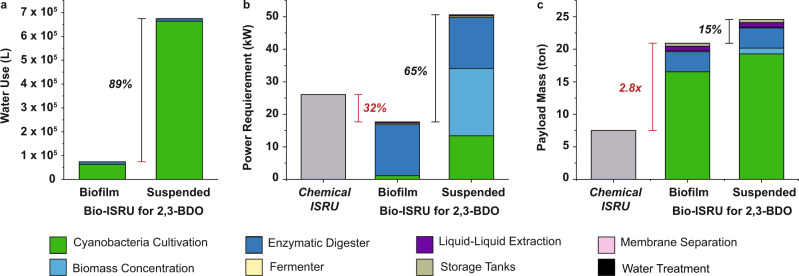
Full process metrics for the bio-ISRU production of 2,3-BDO. **a** Water use, **b** power requirement, and **c** payload mass accounting for all unit operations in the bio-ISRU for 2,3-BDO production using either biofilm or suspended cyanobacteria growth modes. Results are based on 10 tons of 2,3-BDO produced over 500 sols. Chemical ISRU (DRA 5.0 O_2_ only strategy) shown in gray. Source data are provided as a Source Data file.

Holistic analysis of the process was performed to determine water requirement, power use, and total payload mass for the bio-ISRU for 2,3-BDO production (Fig. 6). Cyanobacteria biofilm growth requires 89% less water, 65% less power, and 15% less payload mass than suspended growth. The stark difference in power use (17.64 vs. 50.62 kW) comes from the elimination of mixing during biofilm growth (26% of the power usage) and the removal of the biomass concentration unit operation (41% of the power usage) even with reduced power demand due to reduced Martian gravity. If the energy for water harvesting is included in the calculations, biofilm growth requires 8.5-fold less power than suspended growth.

When compared to the DRA 5.0 O_2_ only strategy, the bio-ISRU for 2,3-BDO production using cyanobacteria biofilm growth requires 2.8-fold greater payload mass from Earth, yet has a 32% lower power requirement (Fig. 6, Table 1). Almost 80% of the biofilm bio-ISRU payload mass comes from cyanobacteria cultivation, due to the large mass of glucose required to feed *E. coli* to produce the 10 tons of 2,3-BDO. Most of the biofilm growth payload mass comes from the cotton material (82%) (Fig. 4b). Of note, the bio-ISRU evolves O_2_, which is sufficient for microbial 2,3-BDO production (~12 tons) and rocket launch (19.6 tons), leaving 43.81 tons of excess O_2_ over 500 sols_._ The excess O_2_ can be used for subsequent aircraft launches or crew life support systems. For context, a crew of 6 consumes 2 tons of O_2_ for life support (e.g. breathing) over the course of 500 sols^2^.

### Bio-ISRU optimization overview

Although the bio-ISRU produces sufficient oxygen for rocket launch and even excess oxygen for use in other applications, it is not competitive to the proposed DRA 5.0 O_2_ only strategy in terms of payload mass (13.42 tons heavier). We put forth a series of model-guided biological and materials improvements that should ultimately render the bio-ISRU competitive to the DRA 5.0 O_2_ only strategy in terms of payload mass with a significantly lower power requirement, while still emitting more than 20 tons of excess oxygen.

### Bio-ISRU biological optimization

To determine engineering targets for improving the bio-ISRU, we focused on three biological strategies: (1) increasing cyanobacterial biomass productivity, (2) increasing glucose yield in the enzyme digester, and (3) increasing 2,3-BDO yield in the fermenter.

**Fig. 7:**
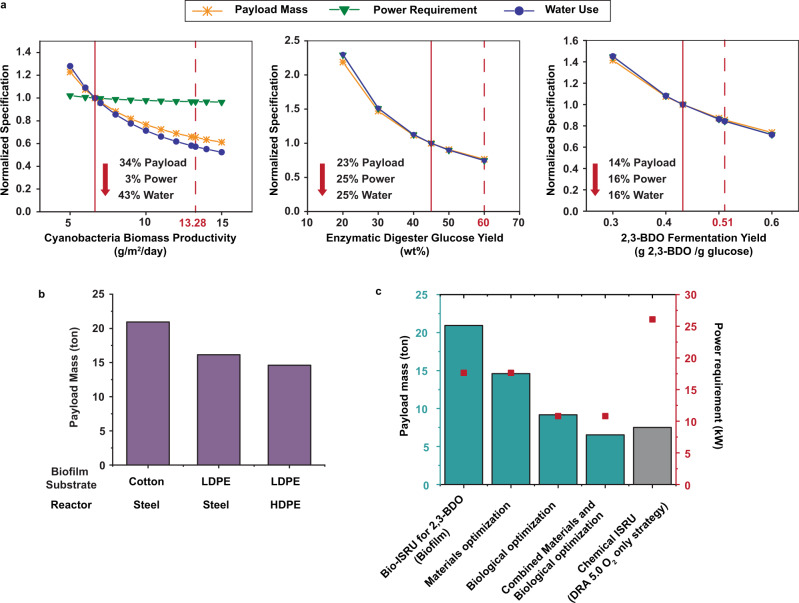
Optimization of the bio-ISRU for 2,3-BDO production. **a** Biological optimization of the bio-ISRU. Payload mass, power requirement, and water use can be reduced by optimizing: Left: cyanobacteria growth productivity, Center: the enzymatic digester glucose yield, and Right: 2,3-BDO fermentation yield from glucose. Solid vertical lines are the productivity and yields of the bio-ISRU for 2,3-BDO production using state-of-the-art technology. Dashed vertical lines are the productivity and yields of the biologically optimized bio-ISRU. **b** Materials optimization of the bio-ISRU. Payload mass changes when switching the material used for the biofilm substrate, from cotton to low-density polypropylene (LDPE), and the reactors, from steel to high-density propylene (HDPE). **c** Summary of payload mass and power requirement reduction of the biofilm-based bio-ISRU for 2,3-BDO as it is optimized. The DRA 5.0 O_2_ only strategy is the mass of shipping CH_4_ from Earth and using SOCE to produce O_2_ from Martian CO_2_ (ref. ^2^). Source data are provided as a Source Data file.

First, we focused on improving cyanobacterial biomass productivity (6.64 g/m^2^/day), as 80% of the payload mass comes from the cyanobacterial growth system. Using process modeling, we analyzed water, power, and payload mass requirements for cyanobacterial growth productivities between 5 and 15 g/m^2^/day (Fig. 7a). Improving productivity to 13.28 g/m^2^/day reduces water usage by 43% and payload mass by 34%. As expected, it only reduced the power consumption by 3%, as the enzyme digester dominates power consumption, and is relatively unchanged by cyanobacterial productivity. Increasing cyanobacterial biomass productivity could be achieved by (a) improving light input by supplementing sunlight with artificial lighting, (b) improving cyanobacteria photosynthetic efficiency, e.g. by utilizing the wider range of the light spectrum that reaches Mars, or (c) increasing the growth rate of cyanobacteria by adapting *A. platensis* to grow faster at 25 °C (35 °C optimal) or using a cyanobacterial species with faster growth rate at low temperatures. If artificial lighting is used to double the photon flux, productivity is increased by 55% to 10.27 g/m^2^/day. However, this adds 190 tons of payload mass and 1150 kW of power due to the large land footprint of the cyanobacterial growth operation based on calculations for solar-powered artificial lighting^62^. Integration of organic light emitting diodes (LED)^63^ powered by photovoltaics^64^ into the dome surrounding the process could provide a lightweight, future strategy for increasing light flux to the cyanobacteria, though the technology requires further developments. Improving cyanobacteria photosynthetic efficiency (*α*, mol of carbon fixed/mol of photons) may be a more viable strategy. The theoretical maximum for *α* is 0.125, i.e. 1 mol of carbon fixed/8 mol of photons. In the model, *α* is set to 0.061, 49% of the theoretical maximum. To double biomass productivity, *α* would need to reach 0.117, or 93% of theoretical maximum. Although improving the photosynthetic efficiency of autotrophic organisms has been difficult^65^, promising strategies, such as RuBisCo overexpression and reducing the size of the light harvesting antennae, have led to improvements in cyanobacterial photosynthesis rate and biomass productivity, respectively^66^.

Second, we looked at increasing the enzyme loading or implementation of undigested biomass recycle stream to increase the glucose yield in the enzyme digester. As modeled, the enzymatic digestion has a 45% yield by weight of total biomass. If the enzymatic digester reached 60 wt% of total digested biomass (Fig. 7a), the payload mass, water requirement, and power use of the entire process would be reduced by ~25%. Although we focus on sugar release, cyanobacteria can accumulate fatty acids to 40–45% of DCW^67^ and in the future, those fatty acids could be extracted and fed to *E. coli*.

Third, the *E. coli* yield of 2,3-BDO from glucose has more limited room for improvement as it is already at 80% of the theoretical maximum (0.432 g/g). Even so, reaching 95% of theoretical yield (0.51 g/g) would reduce water, power and mass requirements by ~16% for all metrics (Fig. 7a). Collectively, increasing cyanobacterial biomass productivity to 13.28 g/m^2^/day, the enzymatic digester yield to 60 wt%, and fermentation yield to 0.51 g of 2,3-BDO/g of glucose, reduces the payload mass of the bio-ISRU to 9.17 tons, just 22% higher than the DRA 5.0 O_2_ only strategy. The 56% payload mass reduction comes from a 69% reduction in cyanobacterial farm size.

### Bio-ISRU materials optimization

To further reduce the bio-ISRU payload mass, we evaluated alternative materials for the cyanobacterial biofilm growth substrate, the microbial fermenter, and other units of operation. The biofilm growth substrate accounts for 64% of the total payload mass of the original bio-ISRU model. Replacing the cotton-like material with a similar density to the LDPE used for the PBR bags reduces the original bio-ISRU payload mass by 23% to 16.15 tons. In the original bio-ISRU model, steel was chosen as the material for the microbial fermenter and other unit operations based on Earth requirements, including ease of sterilization and structural stability. On Mars, the lower gravity should allow for better performance of lighter weight materials. For example, using fermenters made of aluminum or high-density polyethylene (HDPE) would further reduce process mass to 14.98 and 14.60 tons, respectively, if implemented along with the lighter biofilm growth substrate (Fig. 7b).

### Fully optimized Bio-ISRU process analysis

By incorporating the proposed biological and materials improvements, the optimized bio-ISRU will produce 10 tons of 2,3-BDO over 500 Sols, evolving sufficient oxygen for fermentation and rocket launch, and leaving 20.35 tons to support other aspects of Martian exploration. The optimized bio-ISRU requires 38% less power and has a 13% lower payload mass than the DRA 5.0 O_2_ only strategy (Fig. 7c). Further, subsequent bio-ISRU missions will require only 3.73 tons of resupply payload mass, which is less than the needed methane resupply for the DRA 5.0 O_2_ only strategy (6.5 tons). Under Earth conditions, the lifetime of most process components is 10–20 years^68^, and while Martian conditions will likely shorten this lifetime, most process components can be reused over multiple missions. If nutrients and butanol solvent can be successfully recovered, the only resupply needed will be the biofilm growth substrate, which has a lifetime of 1–2 years^40^, and the digestion enzymes, which, as modeled, are only shipped for a single 500 Sol mission.

## Discussion

Taking into account the differences between Mars and Earth will be pivotal to developing efficient technologies for the ISRU production of fuel, food, and chemicals on Mars. By keenly considering these differences, we propose short-chain diols as Martian rocket propellants as they have higher heating values and specific impulses sufficient to launch a human MAV from the surface of Mars into low Mars orbit. Using state-of-the-art technology, we show the feasibility of a biological process to produce 2,3-BDO on Mars and evolve clean O_2_ as a byproduct, which in addition to rocket launch, could help sustain other aspects of human colonization. Using state-of-the-art technology, the bio-ISRU consumes 32% less power than the DRA 5.0 O_2_ only strategy, and when the biological and materials optimizations are realized, the bio-ISRU will consume 59% less power and have a 13% lower payload mass from Earth. Further, the bio-ISRU process will evolve more than 20 tons of oxygen, which can be used for Mars colonizing missions. Importantly, the bio-ISRU design allows its repurposing from propellant production to chemical and food production by simply swapping the engineered heterotrophic microbe. Thus, tackling the biological and materials challenges presented in this work has the potential to enable future human presence beyond Earth to become a reality.

Based on 2,3-BDO specific impulse (*I*_sp_), only 8.4 tons of rocket propellant are needed to launch the MAV from the surface of Mars into Mars low orbit. Nevertheless, we run our process design conservatively, targeting 10 tons of rocket propellant, which is an overestimate of the rocket fuel mass needed for MAV launch. This allows us to buffer for (1) potentially lower experimental 2,3-BDO specific impulse (up to ~383 s), and (2) variability in Martian environmental conditions, such as dust storms limiting growth, start-up time and seasonal light changes. Thus, the results of our process analysis do not hinge on the calculated theoretical *I*_sp_ of 2,3-BDO. As experimental specific impulse values for diols become available, the analysis can be re-run to obtain a more accurate rocket payload mass.

Reducing the number of unit operations or recycling of nitrogen and phosphorus could further improve the bio-ISRU metrics. For example, the enzymatic digestion step could be completely eliminated by engineering cyanobacteria to not store glycogen, but instead secrete glucose^69^ or sucrose^70^ directly to the media. Such an approach would eliminate the enzymatic digestion step and the need to ship digestive enzymes from Earth. In the biologically optimized scenario, cyanobacterial growth will consume 1.19 kg of nitrogen and 0.143 kg of phosphorus per day. A 3-day supply of nutrients, giving ample time for nitrogen and phosphorus to recycle through the enzyme digester, fermenter, and water treatment unit operations could reduce diammonium phosphate and ammonia requirement from 1.23 to 0.004 tons (3.6 kg). We do not include this process optimization in our calculations as the proposed biological and materials improvements result in an energy requirement and payload mass that is lower than the DRA 5.0 O_2_ only strategy.

Current NASA Planetary Protection guidelines prohibit the sending of microbes to the surface of Mars to protect it from contamination by Earth life^71^. As shown in this work, biotechnology applications on Mars have the potential to provide distinct advantages over chemical processes, including chemical production and oxygen generation. Towards Martian bio-ISRU approaches, physical containment barriers, as well as engineered biological containment strategies, will need to be implemented and fully tested on Earth before launching a mission to Mars. Engineering of kill switches and auxotrophic strategies to prevent microbes from surviving outside the reactor is an active area of research^72^. Integrating Mars-specific features may lead to the development of microbes with a lower risk for Mars contamination.

## Methods

### Theoretical *I*_sp_ calculation

Theoretical *I*_sp_ values for 1,2-ethanediol, 1,3-PDO, 1,3-BDO, 2,3-BDO, and butanol were calculated using Eq. (3). Specifically, theoretical values for specific impulse (*I*_sp_) were calculated assuming complete combustion and that all stored chemical energy in the fuel is converted to kinetic energy, allowing a simplified calculation of exhaust velocity. Thus, the exhaust velocity, *v*_e_, could be estimated as shown in Eq. (2). As the *I*_sp_ values were used for comparison only and all compared fuels are hydrocarbons, meaning they have similar combustion temperatures and exhaust compositions if we assume complete combustion. Thus the calculation of *I*_sp_ was approximated using Eq. (3), where *v*_e_ is the exhaust velocity, *g* is Earth’s gravitational acceleration, Δ*H* is the heat of combustion for the fuel of interest, *n*_H2O_ is the number of moles of H_2_O produced from complete combustion of 1 mole of fuel, Δ*H*_vap_ is the heat of vaporization of water, and *m*_ex_ is the molar mass of the exhaust determined based on complete combustion of the fuel of interest.

1 $${I}_{{{\mathrm {sp}}}}=\frac{{v}_{{\mathrm {e}}}}{g}$$

2 $${v}_{{\mathrm {e}}}=\sqrt{\frac{2\left(\triangle H-\triangle {H}_{{{\mathrm {vap}}}}\right)}{{m}_{{{\mathrm {ex}}}}}}$$

3 $${I}_{{{\mathrm {sp}}}}=\frac{\sqrt{\frac{2\left(\triangle H-{n}_{{\mathrm {H2O}}}\triangle {H}_{{{\mathrm {vap}}}}\right)}{{m}_{{{\mathrm {ex}}}}}}}{g}$$

### Ideal rocket equation calculations

The ideal rocket equation (Eq. (4)) was used to calculate the amount of propellant (1,2-ethanediol, 1,2-PDO, 1,3-BDO, 2,3-BDO) required for a rocket launch. Using the assumptions that *d*_v_ (required change in velocity to get to orbit), *g* and *m*_e_ (mass of empty MAV) were constant, we calculated the ratio of fuel needed based on the *I*_sp_ of the proposed propellants when compared to methane. Specifically, *d*_v_ = change in velocity of the rocket (5625 m/s, DRA 5.0 addendum 1, p. 105); *I*_sp_ = theoretical specific impulse of the propellant; *g* = Earth’s gravitational acceleration (9.81 m/s^2^); *m*_f_ = mass of the rocket full; *m*_e_ = mass of the rocket empty = mass of MAV (8.542 tons); mass of propellant = *m*_f_−*m*_e_.

4 $${d}_{{\mathrm {v}}}={I}_{{{\mathrm {sp}}}}\times g\times {{\mathrm {ln}}}\left(\frac{{m}_{{\mathrm {f}}}}{{m}_{{\mathrm {e}}}}\right)$$

### Martian rocket propellants experimental calculations

Experimental yields of 1,2-PDO, 1,3-BDO and 2,3-BDO were determined as follows. The 1,2-PDO yield (0.178 g/g glucose) was calculated from the reported 1,2-PDO molar yield (0.422 mol 1,2-PDO/mol glucose)^27^. The 1,3-BDO yield (0.11 g/g glucose) was calculated from the reported glucose consumed during fed-batch fermentation (83.05 g/L) and the reported 1,3-BDO titer (9.05 g/L)^28^. The 2,3-DBO yield (0.432 g/g glucose) was calculated from the reported glucose consumed during fed-batch fermentation (171 g/L) and the reported 2,3-BDO titer (73.8 g/L)^29^. With respect to 2,3-BDO productivity (1.17 g/L/h), since the fermenter in the bio-ISRU process will be run continuously and not as a batch, the 2,3-BDO productivity used in the model was the productivity during the stationary growth phase of batch fermentation^29^. Stationary growth phase was taken between the 24 and 48 h time points, during which 28 g/L of 2,3-BDO were produced.

### Cyanobacterial growth model

Cyanobacterial growth model (Eq. (5)) adapted from Karemore et al. ^41^ describes *A. platensis* biomass productivity (*P*_Biomass_) assuming growth is light limited. The model takes into account carbon fixation and respiration when calculating productivity. Model parameters can be found in Supplementary Table 1.

5 $${P}_{{{\mathrm {Biomass}}}}=F({\alpha E}_{{\mathrm {k}}}\gamma \,{{\mathrm {ln}}}{{\lfloor }}\frac{{E}_{{\mathrm {k}}}+{E}_{0}}{{E}_{{\mathrm {k}}}+{E}_{0}{e}^{-{kD}}}{{\rfloor }}\frac{{t}_{1}}{D}-{R}_{0}{C}_{{\mathrm {c}}}\gamma {t}_{2})$$

### Process requirements

Unit operation specification can be found in Supplementary Note 1.

The required O_2_:fuel ratio is the mass of O_2_ required for complete combustion of the fuel, on a per mass basis.

The water requirement of the system was calculated based on the water usage of the cyanobacterial growth unit, the enzyme digester, and the fermenter. For suspended growth, water use was based on the sizing and spacing of the PBRs, which led to a requirement of 41.47 L/m^2^ land area. For biofilm growth, water use was based on 2.18 L/m^2^ of growth substrate area required in the previous literature^45^. The water requirement for the digester and fermenter was based on the calculated flowrate out of the cyanobacterial concenter (or from the growth unit in the case of biofilm growth) and the residence time of the digester (48 h) and fermenter (72 min). No water loss was accounted for and 100% of the required water was recycled.

The power requirement for the suspended cyanobacteria growth came from culture mixing and pumping. Mixing was assumed to be directly proportional to gravity with a power input of 52 W/m^3^ of culture media while pumping power was taken from literature at 0.058 kWh/m^3^ with the volume corresponding to the flow rate coming out of the cyanobacterial growth operation^68^. The power requirement for the biofilm cyanobacteria growth came only from pumping required for nutrient resupply and was based on the pilot scale value of 0.038 W/m^2^ culture area^45^. The power requirement for the cyanobacteria concentrator came from the cross-flow filtration unit; it was assumed to be 5 kWh/m^3^ of volume processed based on the previous literature^48^. The power requirement for the digester, fermenter, and LLE units was calculated based on the power required for reactor agitation via impeller mixing. Since Mars has decreased gravity compared to Earth, the power requirement for mixing would be lower. As a conservative estimate, we estimated a 25% reduction in power requirement, thus making the mixing requirement 1.5 kW/m^3^ of reactor volume^73^. Power requirement for the membrane separation unit was calculated based off the energy model developed in previous literature for membrane separation of 2,3-BDO from butanol^74^, the same system used in our process model.

The payload mass for the cyanobacterial growth was comprised of the mass of the growth substrate, the mass of the support frame, and the mass of the nutrients required for cyanobacterial growth. The growth substrate mass for suspended growth was based on the dimensions of the PBR and a 0.3 cm-thick LDPE material. In the case of biofilm growth, the mass was based on the total growth substrate area and a 0.3 cm-thick cotton material. The required nutrient mass was based on the requirement of 0.029 and 0.002 moles of nitrogen and phosphorous required per mole of biomass. Phosphorous was entirely provided by diammonium phosphate, which also provides two moles of nitrogen. The rest of the nitrogen was provided by ammonia. Enough nitrogen and phosphorous was shipped to supply the process during the entire 500 day mission with 20% excess, assuming no nitrogen or phosphorous recycling. In addition, trace elements were provided for a media composition of 1 g/L NaCl, 0.03 g/L CaCl_2_, 1 g/L K_2_SO_4_, 0.008 g/L MgSO_4_, 0.08 g/L EDTA, 0.01 g/L FeSO_4_, 0.00023 g/L MnCl_2_, 0.00011 g/L ZnSO_4_, and 0.00003 g/L CuSO_4_ (ref. ^35^). The required mass of the polyvinylchloride (PVC) support frame was estimated based on the 78 tons/hectare of steel support structure used in pilot-scale suspended growth^68^. Frame mass was corrected for Martian gravity which will reduce frame support needed by reducing the weight of the algal cultivation reactors. Mass for biofilm growth was further reduced due to the reduced water content that makes the total mass of the biofilm reactors 11% of the suspended system. The total mass needed was based on the volume of the support frame and a PVC density of 1.38 tons/m^3^. The cyanobacteria concentrator payload mass was calculated based on requiring 155.61 m^2^ of organic membrane housed in a steel cylinder 2 m long with a 1 m radius. The enzyme reactor payload mass was based on a steel vessel with a 2.5 height to radius ratio, with 20% excess volume to the volume required to reach the desired residence time. In addition, the mass of enzymes needed for biomass digestion was included in this calculation. Here we assumed enzyme concentration of 1 g/L lysozyme, 1 g/L amylase, and 1 g/L glucoamylase. Enough enzymes were shipped to allow the enzymes to be replaced every 10 days. The fermenter payload mass was based on a steel vessel with a 2.5 height to radius ratio, with 20% excess volume to the volume required to reach the desired residence time of 72 min. The residence time was calculated based on the inflow rate of glucose and the glucose consumption rate in order to consume all fed glucose, i.e. glucose input divided by the product of 2,3-BDO productivity times yield. The LLE reactor payload mass was based on a steel vessel with a 2.5 height to radius ratio, with 20% excess volume to the volume required to reach the desired residence time. In addition, the mass of butanol needed for extraction was included in this calculation. The mass of butanol was modeled based on a pure butanol input flow rate of 0.001 L/min, this input rate gave us our desired 95% 2,3-BDO purity. The membrane separator payload mass was calculated based on requiring 0.5 m^2^ of polydimethylsiloxane/polyvinyledenefluoride membrane housed in a steel cylinder 0.5 m long with a 0.007 m radius. The payload mass of 2,3-BDO and O_2_ storage tanks were calculated based off 16 and 28 m^3^, respectively, composed of 0.006 m-thick HDPE. The water treatment payload mass was calculated based on requiring 13.1 m^2^ of thin-film composite membrane housed in a steel cylinder 0.5 m long with a 0.036 m radius.

Excess O_2_ production was calculated based on 1 mole of O_2_ produced for every mole of CO_2_ fixed into biomass. O_2_ was consumed in the fermenter at a rate of 0.12 mol O_2_/L/h at a biomass concentration of 6 g/L^75^.

### Process modeling

Calculations were implemented in a Python-based process model to determine process specifications for a given cyanobacteria biomass productivity (g/m^2^/day), enzyme digester yield (g glucose/g biomass), fermentation yield (g 2,3-BDO/g glucose), density of biofilm growth substrate (g/cm^3^), and density of reactor material (g/cm^3^). The process model uses these inputs to first determine the land area of cyanobacterial culture that is needed to produce 10 tons of 2,3-BDO in 500 days. Then, the model calculates the power, water, and mass usage for the entire process and outputs those calculations, as well as the land calculation, into an excel file. To perform the calculations, the model uses stoichiometric calculations for the cyanobacteria growth through 2,3-BDO production in the fermenter. The resulting water and 2,3-BDO stream is then combined with a butanol stream and thermodynamic equilibrium was calculated using the Phasepy python package^57^. The butanol-rich stream was then fed into a membrane separator, with the compositions of the 2,3-BDO and butanol-rich streams calculated using published differential equations^56^. To implement a recycle loop, a non-linear equation solver (‘root’ solver from the Scipy package^76^) was used to determine final stream compositions in the separations module. This workflow was used to generate the data presented in Figs. 6 and 7 by changing input parameters.

### Flux balance analysis

Theoretical yields of the analyzed compounds were calculated using the COBRA toolbox v3.0 in Matlab^30^. Engineered pathways for 1,2-PDO^27^, 1,3-BDO^28^, and 2,3-BDO^29^ were added to the genome scale model for *E. coli* MG1655, iML1515^31^. Endogenous metabolites were used when available and pathways started from lactic acid, 3-hydroxybutyryl-CoA, and α-acetolactone for 1,2-PDO, 1,3-BDO, and 2,3-BDO, respectively. For engineered 1,2-PDO biosynthesis, reactions present in the base model which produce methylglyoxal were removed (simulated gene deletion) to ensure that 1,2-PDO flux was calculated through the engineered pathway and not confounded by the native pathway. Glucose uptake rate (EX_glc_D_e) was fixed at −10, and O_2_ uptake rate (EX_o2_e) was fixed at -15 for all pathway simulations. Theoretical yields were obtained using the ‘optimizeCbModel’ function with the objective function set for the exchange reaction of the final product. The reactions and metabolites added for each pathway can be found in Supplementary Note 2.

### Reporting summary

Further information on research design is available in the Nature Research Reporting Summary linked to this article.

## Supplementary information

Supplementary Information

Reporting Summary

## Data Availability

Data supporting the findings of this work are available within the paper and its Supplementary Information files. A reporting summary for this Article is available as a Supplementary Information file. Source data are provided with this paper.

## Source data

Source Data

## Code availability

Code used for propellant property calculations (O_2_/propellant ratio, lower heating value), process modeling, FYI: specific impulse calculations and rocket propellant masss calculations are in Source data (Fig. 2c) and flux balance analysis of 1,2-PDO, 1,3-BDO, and 2,3-BDO is available at GitHub [https://github.com/PPYLab/bioISRU].
