# Supplementary material for: Designing the bioproduction of Martian rocket propellant via a biotechnology-enabled in situ resource utilization strategy
Source: Nat Commun. 2021 Oct 25;12:6166. doi: 10.1038/s41467-021-26393-7 (PMC8546151; doi:10.1038/s41467-021-26393-7)
Supplement: Supplementary file 1 — Supplementary Information [file 41467_2021_26393_MOESM1_ESM.pdf]

Designing the bioproduction of Martian rocket propellant *via* a biotechnology-enabled *in situ* resource utilization strategy

Kruyer *et al.*

Supplementary Table 1. Cyanobacterial growth model parameters.

Model parameter	Parameter definition	Parameter value biofilm	Parameter value suspended	Source
P	Biomass productivity	6.54	6.6	
F	Ratio of light absorption area to land area	1.84	1.84	This work
α	Photosynthetic efficiency	0.061 mol C x mol photons^{-1}		[1]
E_K	optimal photon flux[a]	161.01 µmol photons x m^{-2}*s^{-1}		[1], this work
E_0	photon flux at reactor surface on Mars	142.8 µmol photons x m^{-2}*s^{-1}		[2]
k	light penetration parameter	8750 m^{-1}	175 m^{-1}	[1]
D	reactor diameter	0.00025 m [3]	0.04 m [4]	This work
γ	g biomass per mole of carbon fixed	22.2 g DCW x mol C^{-1}		[4]
t_1	Light cycle time (12 hrs photosynthesis)	43200 s	43200 s	[1]
R_0	cellular respiration rate[a]	0.001395 µmol C x mg Chl^{-1} x min^{-1}		[1], this work
C_c	areal chlorophyll content	135 mg Chl x m^{-3}	18000 mg Chl x m^{-3}	[1]
t_2	total day cycle time (24 hrs)	84,600 s	84,600 s	[1]

[a] At 25°C

Supplementary Table 2. Temperature effect on cyanobacteria biofilm productivity.

Temperature (°C)	Productivity ($g/m^2/day$)	% Increase over 25°C	Added heat (kJ)
25	6.64	0%	0
30	7.44	12%	2.32×10^6
35	8.05	21%	4.64×10^6

Supplementary Table 3. Light effect on cyanobacteria biofilm productivity.

Temperature (°C)	Light flux	Productivity ($g/m^2/day$)	% Increase over Mars base case
25	142.8	6.64	0%<br>Mars base case
	150	6.88	4%
	175	7.65	15%
	200	7.34	11%
	225	8.97	35%
	250	9.54	44%
	285.6	10.27	55%
30	142.8	7.44	12%
	150	7.75	17%
	175	8.74	32%
	200	9.65	45%
	225	10.5	58%
	250	11.28	70%
35	142.8	8.05	21%
	150	8.42	27%
	175	9.61	45%
	200	10.74	62%
	225	11.8	78%
	250	12.79	93%

Supplementary Table 4. Photosynthetic efficiency effect on cyanobacteria biofilm productivity at 25°C.

Photosynthetic efficiency, α (mol C/mol photon)	Productivity ($g/m^2/day$)	% Increase over Mars base case	% of theoretical	Efficiency
0.061	6.64	0%	0.488	0%
0.07	7.72	16%	0.56	15%
0.08	8.92	34%	0.64	31%
0.09	10.11	52%	0.72	48%
0.1	11.31	70%	0.8	64%
0.11	12.5	88%	0.88	80%
0.125	14.3	115%	1	105%

Supplementary Table 5. Einsteins to photon flux conversion

	einsteins	Photons
**Earth**	3.32E-04	2.00E+20
**Mars**	1.43E-04	8.60E+19

Supplementary Note 1. Unit operation specifications.

Material density

- Stainless steel = 7.85 g/cm^3
- Aluminum = 2.7 g/cm^3
- Low density polyethylene (LDPE) = 1 g/cm^3
- High density polyethylene (HDPE) = 1 g/cm^3
- Cotton = 1.55 g/cm^3
- Polyvinylchloride (PVC) = 1.38 g/cm^3

Suspended growth PBR

- 4.5 cm thick, 1 m tall, 45 m wide
- PBR material: 0.3 mm thick LDPE

Biofilm growth substrate

- 1 m tall
- Growth substrate material: 0.3 mm thick cotton

Biomass concentrator

- Reactor material = 6 cm thick steel

Enzyme digester

- 20% headspace on volume determined by flow rate and desired residence time
- Rector height:reactor radius ratio = 2.5
- Reactor material = 6 mm thick stainless steel
- Reactor residence time = 48 hours

***E. coli* fermentation**

- 20% headspace on volume determined by flow rate and desired residence time
- Rector height:reactor radius ratio = 2.5
- Reactor material = 6 mm thick stainless steel
- Reactor residence time = 72 minutes

Liquid-liquid equilibrium extraction

- 20% headspace on volume determined by flow rate and desired residence time
- Rector height:reactor radius ratio = 2.5
- Reactor material = 6 mm thick stainless steel
- Reactor residence time = 60 minutes

Membrane separation unit

- Reactor material = 6 cm thick stainless steel
- Membrane = polydimethylsiloxane/polyvinyledenefluoride

Storage tanks

- 2,3-BDO storage: 16 m^3 storage tank made of 6 cm thick HDPE
- O_2 storage: 28 m^3 storage tank made of 6 cm thick HDPE

Supplementary Note 2. Reactions and metabolites in engineered pathways.

1,2-Propanediol

Metabolites added:

Lacoyl-CoA: lac_coa_c

Reactions added:

Lactoyl-CoA transferase: lac__D_c + accoa_c -> lac_coa_c + ac_c

Lactaldehyde dehydrogenase: lac_coa_c + nadh_c -> lald__D_c + nad_c + coa_c

Reactions removed:

MGSA: dhap_c -> pi_c + mthgxl_c

AACTOOR: o2_c + aact_c + h2o_c -> nh4_c + mthgxl_c + h2o2_c

1,3-Butanediol

Metabolites added:

3-hydroxybutyraldehyde: 3hbald_c

1,3-butanediol, cytosolic: 13bdo_c

1,3-butanediol, extracellular: 13bdo_e

Reactions added:

Butyraldehyde dehydrogenase: 3hbcoa_c + nadh_c -> 3hbald_c + nad_c + coa_c

Butyraldehyde dehydrogenase: 3hbald_c + nadh_c -> 13bdo_c + nad_c

1,3-BDO transport: 13bdo_c <=> 13bdo_e

1,3-BDO exchange: 13bdo_e <=>

2,3-Butanediol

Metabolites added:

R-acetoin: acetoin_c

2,3-butanediol, cytosolic: 23bdo_c

2,3-butanediol, extracellular: 23bdo_e

Reactions added:

α-Acetolactate decarboxylase: alac__S_c -> acetoin_c','co2_c

2,3-Butanediol dehydrogenase: acetoin_c + nadh_c -> 23bdo_c + nad_c

2,3-BDO transport: 23bdo_c <=> 23bdo_e

2,3-BDO exchange: 23bdo_e <=>

Supplementary references

1. Karemore, A., Yuan, Y., Porubsky, W. & Chance, R. Biomass and pigment production for *Arthrospira platensis* via semi-continuous cultivation in photobioreactors: temperature effects. *Biotechnol. Bioeng.* **117,** 3081-3093 (2020).
2. Verseux, C. et al. Sustainable life support on Mars – the potential roles of cyanobacteria. *Int. J. Astrobiol.* **15,** 65-92 (2015).
3. Li, T., Piltz, B., Podola, B., Dron, A., de Beer, D., Melkonian, M. Microscale profiling of photosynthesis-related variables in a highly productive biofilm protobioreactor, *Biotechnol Bioeng*, **113**, 1046-1055 (2015).
4. Clippinger, J.N. & Davis, R.E. Techno-economic analysis for the production of algal biomass via closed photobioreactors: future cost potential evaluated across a range of cultivation system designs. *National Renewable Energy Laboratory* NREL/TP-5100-72716 (2019).
